# Genome-Wide SNP Characterisation of Three Kazakh Sheep Breeds: Kazakh Fat-Tailed Coarse-Wool, Degeres, and Etti Merino

**DOI:** 10.3390/ani16172754

**Published:** 2026-09-02

**Authors:** Kirill Yanin, Rakhim Kanat, Iskander Isgandarov, Dastanbek Baimukanov, Zagipa Sapakhova, Tatyana Ulyanova, Alexandr Kovalchuk, Vadim Ulyanov, Indira Beishova, Nurzhan Zhumadillayev, Narzhan Zhumadillayev, Daryn Janayev, Anastasiya Yanina, Malika Shamekova

**Affiliations:** 1 Tanir Research Laboratory, Almaty 050040, Kazakhstan; k.yanin@ipbb.kz (K.Y.);; 2 Institute of Plant Biology and Biotechnology, Almaty 050010, Kazakhstandbaimukanov@mail.ru (D.B.);; 3 Zhangir Khan West Kazakhstan Agrarian Technical University, Oral 090009, Kazakhstanvadimkst19@gmail.com (V.U.);

**Keywords:** *Ovis aries*, Kazakhstan, Degeres, Etti Merino, Kazakh fat-tailed coarse-wool, SNP genotyping, population structure, genetic diversity, ADMIXTURE, breed conservation

## Abstract

Kazakhstan’s sheep portfolio underpins much of the country’s mutton and wool production, yet several of its principal breeds remain genomically uncharacterised. The aim of this study was to characterise the genomic diversity, population structure, and global phylogenetic placement of three economically important Kazakh breeds and to determine whether they constitute separate gene pools requiring independent management. We present the first genome-wide SNP characterisation to include the Degeres (DE), the Etti Merino (EM), and the Kazakh fat-tailed coarse-wool (KKG) breeds simultaneously. A total of 1497 animals (DE = 354, EM = 642, KKG = 501) sampled across seven production households were genotyped and, after quality control, analysed at 42,279 SNPs, of which 22,766 LD-pruned markers were used for principal component analysis and AMOVA. We applied principal component analysis (PCA), pairwise *F*_ST_, analysis of molecular variance (AMOVA), neighbour-joining phylogenetics, model-based ancestry estimation (ADMIXTURE), and Hill-number diversity profiling, and projected the breeds against the global Ovine SNP50 HapMap panel (74 reference breeds, 2819 animals; 37,685 shared SNPs). All three breeds retained uniformly high within-breed diversity (expected heterozygosity 0.413–0.417) with fixation indices at or near zero. AMOVA partitioned 94.03% of variance within breeds (Φ_ST_ = 0.060, *p* < 0.001). PCA, phylogeny, and ADMIXTURE concordantly resolved three breed-specific clusters at *K* = 3, with a maximum interbreed *F*_ST_ of 0.038 within the study dataset. Against the global panel, EM was genetically closest to Merino and Merino-derived reference breeds (pooled *F*_ST_ = 0.017) and substantially more distant from Southwest Asian sheep (*F*_ST_ = 0.045), whereas DE and KKG showed the reciprocal pattern (*F*_ST_ = 0.027 and 0.020 to Southwest Asia, 0.052 to the Merino group). DE additionally displayed the heterozygote excess and partial admixture expected of an incompletely consolidated composite. These results delineate three distinct gene pools and carry direct implications for breed management and the conservation of genomic diversity in Kazakhstani sheep.

## Simple Summary

Kazakhstan relies heavily on sheep farming for meat and wool, but several of its major breeds have never been studied at the DNA level. This study looked at three economically important breeds—Degeres, Etti Merino, and Kazakh fat-tailed coarse-wool—using genetic markers spread across the genome in nearly 1500 animals from seven farms. The results show that Etti Merino, despite being raised in Kazakhstan for over a decade, still carries the genetic signature of its Merino ancestors and resembles Merino and Merino-derived sheep more than it resembles neighbouring Asian breeds. Degeres, by contrast, shows signs of still being assembled as a breed, consistent with its known history of crossing multiple breeds together. All three breeds carry high genetic diversity, with almost all of the genetic variation found within each breed rather than between them, meaning none shows signs of inbreeding or genetic bottlenecks. These findings give breeders and policymakers a genomic baseline for managing and conserving these breeds as three genetically distinct populations.

## 1. Introduction

Domestic sheep (*Ovis aries*) were domesticated from wild mouflon (*Ovis orientalis*) some 8000–11,000 years ago. Recent archaeogenomic work places the centre of domestication near central Anatolia and reconstructs a history of repeated post-domestication dispersal and admixture, including a major Bronze Age influx of Western-steppe ancestry, rather than a single radiating wave [1,2,3], and whole-genome resequencing has traced the migration corridors that carried sheep between the Eurasian steppe and the Iranian plateau [4]. Kazakhstan occupies a pivotal position within this history: its steppe and semi-desert landscapes, situated along the ancient Silk Road, have sustained nomadic pastoralism for millennia and produced sheep finely adapted to continental climate extremes and extensive migratory grazing. Sheep farming remains central to the national agricultural economy, supplying the greater part of the country’s mutton and wool and providing the main livelihood of the extensive pastoral regions. Three breeds are of particular economic significance in contemporary Kazakhstan and form the focus of this study: together they span the country’s coarse-wool to fine-wool production range and account for a substantial share of its commercial mutton and wool output.

The Degeres (DE), bred in the foothill zone of south-east Kazakhstan (Almaty region), is a fat-tailed mutton–wool composite of relatively recent origin, formed by crossing local fat-tailed stock with introduced breeds and consolidated through prolonged ‘breeding in itself’ of multi-breed hybrids [5]. It is maintained as two intra-breed types: a semi-fine, meat–wool–greasy type, approved in 1980, derived from three-breed hybrids of Kazakh fat-tailed, Shropshire, and Precoce sheep, and notable as one of the few fat-tailed breeds worldwide to carry a semi-fine fleece (48–58 quality, staple length 12–15 cm, clean-fibre yield 58–62%), and a semi-coarse carpet type of white to light-grey colour, tested in 2009 from Edilbay ewes crossed with semi-fine Degeres [5,6]. At the Madi breeding farm, rams average roughly 95 kg and ewes 56 kg, with greasy fleece weights of 6.5 and 3.9 kg, respectively, and lambs reach 35–38 kg by 4.5 months [5]. Microsatellite surveys classify the breed as ‘Degeres mutton–wool’ and assign it the synthetic, composite origin shared across Kazakh breeds [7].

The Etti Merino (EM) is a meat-oriented Merino derivative, created in 2011 as a meat-and-fine-wool breed oriented toward export production [8]. It was developed by crossing Kazakh fine-wool ewes with German Mutton Merino (Merinofleischschaf) rams, building on the earlier large-framed Sarybulak fine-wool type, and combines high meat productivity and early maturity with fine wool of 22–26 µm (60–64 quality) [8]. Adult ewes weigh 65–75 kg and rams 100–120 kg, lambs reach 35–50 kg by four months at average daily gains of 250–350 g, and the breed is kept year-round in the sharply continental, arid sands of south-east Kazakhstan (Saryesik-Atyrau, Moyinkum), conditions under which few meat–fine-wool breeds elsewhere are maintained [8]. It is the dominant commercial meat breed in specialised production households: its polled (hornless) condition and growth have been characterised in the consolidating population [9], its milk yield exceeds that of comparator meat merinos [10], and its rams are widely used to raise the meat productivity of other fine-wool stock such as the South Kazakh Merino [8].

The Kazakh fat-tailed coarse-wool (KKG) is among the oldest and most widely distributed indigenous breeds of Kazakhstan, shaped predominantly by natural selection under nomadic pastoralism rather than by managed breeding, and concentrated in the western, central, and southern semi-desert and desert-steppe zones where pasture is poor and the climate extreme [11]. It is a dual-purpose breed valued for its adaptability to temperature extremes, long-distance pasture migration, and the quality of its fat reserves: a well-developed fat tail serves as an energy store, the robust skeleton, deep chest, and long legs suit extensive seasonal grazing, and the coarse, heterogeneous wool, diverse in colour, is used chiefly for carpets and felt [11]. Adult ewes weigh 55–70 kg and rams 90–110 kg, with well-developed meat conformation, and lambing rates run at 100–115% [11]. The breed retains high early growth with a slaughter yield of roughly 50–52% under low-input pasture systems, including the cold, continental conditions of central and northern Kazakhstan [11,12], and remains the principal maternal base for introductory crossbreeding programmes aimed at raising mutton output [13].

Genome-wide work has established that the majority of ovine genetic variation resides within rather than between breeds, and that population structure reflects historical migration and breed-formation history more than registry boundaries [1,14,15]. Surveys spanning northern Eurasia, the Iranian plateau, and East Asia have repeatedly resolved geographically coherent clusters, including a distinct fat-tailed and a composite grouping separable from fine-wool types [15,16,17], and indigenous Russian and Central Asian breeds have been shown to partition by wool type and geographic origin [18]. Intercontinental studies of Merino and Merino-derived populations have traced their Iberian origin and shown that a shared Merino ancestry component persists across the breeds derived from them worldwide [19,20]. Kazakhstani Merino-derived stock, however, has never been placed within this framework. For Kazakh sheep more broadly, genomic characterisation remains sparse: earlier microsatellite work separated fat-tailed coarse from fine-wool types [21], and recent SNP-array studies have described subsets of the national portfolio [22,23]. None has included DE or EM, and none has resolved how Kazakhstan’s dominant commercial Merino derivative relates to the indigenous fat-tailed stock with which it shares production systems.

Despite their agricultural centrality, the three breeds have never been characterised together at genome-wide resolution. Earlier work on the Kazakh portfolio has relied on microsatellite panels, which document high within-breed variability, low interbreed differentiation, and a reduced ‘genetic purity’ consistent with the synthetic, reticulate origins of these breeds [7,24]. None, however, has placed DE or EM alongside KKG, and the question of how a Merino-derived breed relates genetically to the indigenous stock with which it is co-managed has not been addressed.

Here we present a genome-wide SNP characterisation of DE, EM, and KKG sampled across seven production households. The aim of the study was to quantify genomic diversity within and between the three breeds, resolve their phylogenetic relationships, assess interbreed admixture and the hierarchical partitioning of variance, and situate Kazakhstani breed structure within the global sheep genomic landscape using published reference populations, in order to establish whether the three constitute distinct gene pools requiring separate management. To these ends, we applied principal component analysis, pairwise *F*_ST_, analysis of molecular variance, neighbour-joining phylogenetics, model-based ancestry estimation, and Hill-number diversity profiling. Three results organise what follows: EM retains a global Merino genomic signature and is genetically closer to Merino and Merino-derived reference breeds than to the Asian populations it neighbours geographically, while DE and KKG resolve among Asian sheep; DE shows the genomic signature of an incompletely consolidated composite; and all three breeds retain uniformly high within-breed diversity, with the great majority of variance partitioned within rather than between breeds. Together, these findings carry direct implications for breed management and the preservation of genomic diversity in Kazakhstani sheep.

## 2. Materials and Methods

### 2.1. DNA Isolation and SNP Genotyping

Genetic material was collected from Kazakh sheep at seven farms across various regions of Kazakhstan: 354 Degeres (two households), 642 Etti Merino (one household), and 501 Kazakh fat-tailed coarse-wool animals (four households). Sampling was carried out for the purpose of DNA extraction only. Plucked hair follicles were taken from each animal, the follicle itself rather than the hair shaft serving as the source of DNA, and no fleece, milk, or other production phenotype was recorded in this study. The sampled animals were nulliparous ewes aged 12–13 months, none of which had been bred or lambed at the time of sampling and none of which was therefore lactating, together with breeding rams aged 2–5 years. Samples were collected in March and April 2025. All animals were maintained under the same regime across the seven farms: extensive grazing on natural pasture, which was their sole feed source. Follicle samples were stored at +4 °C for subsequent use, and were used for DNA extraction using the DNK-Extran2 kit (Syntol, Moscow, Russia), in accordance with the manufacturer’s operating procedures. Quantification was performed using a Qubit 4 fluorometer in conjunction with Qubit dsDNA broad range reagent (Thermo Fisher Scientific, Waltham, MA, USA) to ensure quality control for downstream SNP genotyping. Samples were collected from a total of 1497 sheep and processed to extract DNA and perform SNP genotyping. Genotyping was performed using an OvineSNP50v3_HTS SNP microarray in conjunction with an iScan system (Illumina, San Diego, CA, USA), in accordance with the manufacturer’s provided protocol. Genotypes were determined and exported to PLINK format using the genotyping module of GenomeStudio software v2.0.5 (Illumina, San Diego, CA, USA).

### 2.2. Genotyping Quality Control and Analysis

Raw genotype data were processed using PLINK v1.9 [25,26] with a chromosome set of 26 autosomes. SNPs were filtered to exclude loci with call rates below 90%, minor allele frequency below 1%, and significant deviation from Hardy–Weinberg equilibrium (*p* < 1 × 10^−6^). Individuals missing more than 50% of genotype calls were removed. Following QC, 42,279 SNPs and 1497 animals were retained across the three breeds. Of these markers, 41,292 map to autosomes 1–26, 983 map to the X chromosome, and 4 are unplaced. For the principal component analysis and AMOVA only, the autosomal markers were pruned for linkage disequilibrium using SNPRelate v1.40.0 (snpgdsLDpruning) with the composite-LD estimator, a 500 kb sliding window and a threshold of *r*^2^ < 0.2, together with that function’s default marker filters (monomorphic markers excluded, MAF ≥ 0.005, per-marker missingness ≤ 0.05). Because the sliding window starts at a randomly chosen position, the random seed was fixed (seed = 42) so that the retained marker count is reproducible. This procedure retained 22,766 independent SNPs. All remaining analyses—pairwise *F*_ST_, the neighbour-joining tree, Hill-number diversity profiles, and the per-breed and per-household diversity statistics—were performed on the full 42,279-SNP panel.

Breed- and household-level diversity statistics were computed in PLINK v1.9 on the full 42,279-SNP panel, with allele frequencies estimated separately within each group: observed and expected heterozygosity from --hardy (averaged over markers), the method-of-moments inbreeding coefficient *F* from --het (averaged over animals), and minor allele frequency from --freq (averaged over markers). Sex was not coded in the genotype files, so the 983 X-linked markers were treated as diploid and retained in the --hardy and --freq summaries. Because the cohort includes rams, which are hemizygous on X, this affects *H*_o_, *H*_e_, and MAF slightly, but restricting the calculation to the 41,292 autosomal markers changes the values reported in Table 1 and Table 2 by no more than 0.001. PLINK’s --het restricts its scan to autosomal and unplaced markers, so *F* was estimated from 41,296 markers.

Principal component analysis (PCA) was performed on the pruned SNP set using the SNPRelate package [27] in R v4.4.1. To place the study breeds in global context, genotypes were merged with the ISGC OvineSNP50 HapMap reference panel [1], comprising 2819 animals from 74 breeds. One DE individual did not survive merging with the reference panel, leaving 1496 Kazakhstani animals and 4315 animals in total. Markers whose alleles mismatched between the two array versions were strand-flipped and the merge reattempted, which recovered the 37,685 SNPs common to both platforms. Reference breeds were assigned to geographic subregions on the basis of published breed origin data. The per-breed sample sizes and group assignments of the full reference panel are given in Supplementary Table S2. PCA was then repeated on these 37,685 shared SNPs, with no additional LD pruning. Outlier individuals deviating more than four standard deviations from the mean on PC1 or PC2 in an initial PCA round were excluded prior to the final analysis, leaving 4205 animals.

Pairwise *F*_ST_ was estimated between all breed and household pairs using the Weir and Cockerham [28] estimator, as implemented in SNPRelate. Analysis of molecular variance (AMOVA) was performed using the pegas package v1.4 [29] on a Euclidean distance matrix computed from the pruned genotype matrix, with significance assessed by 1000 permutations. AMOVA was run separately at the breed level and household level to partition variance within and between groups.

A neighbour-joining (NJ) phylogenetic tree was constructed from a pairwise identity-by-state (IBS) distance matrix computed using SNPRelate across all 1497 individuals. The NJ algorithm was applied using the ape package v5.8.1 [30], and the resulting tree was visualised with a circular layout using ggtree v3.14.0 [31], with tips coloured by breed of origin. The same procedure was applied to the merged global dataset across all 4315 individuals using the 36,826 autosomal markers of the merged panel. Because tip adjacency in an individual-level tree of this size is not a reliable indicator of breed affinity, a second neighbour-joining tree was constructed at the population level from Nei-corrected net distances between population means, computed from the same IBS matrix across all 77 populations. That tree is presented in Supplementary Figure S1 and its underlying distance matrix in Supplementary Table S3.

Allelic diversity was characterised using Hill numbers, which provide a unified diversity framework across a continuous order parameter *q* [32]. Diversity profiles spanning *q* = 0 (allelic richness), *q* = 1 (Shannon entropy-based diversity), and *q* = 2 (inverse Simpson concentration) were computed per household using the gl.report.diversity function in the dartR package v2.9.9.5 [33].

Model-based ancestry estimation was performed using ADMIXTURE v1.3 [34]. Two analyses were run. The first was unsupervised and used the three Kazakhstani breeds alone, for *K* = 2 through *K* = 8 with three independent replicates per *K*. The most supported number of ancestral components was assessed from 10-fold cross-validation error and the Δ*K* statistic [35]. The second placed the three breeds against the global panel rather than against one another: a supervised analysis of the merged dataset in which the 2819 reference animals were fixed to the six continental groups of Supplementary Table S2 (Africa, *n* = 145; Americas criollo, *n* = 210; Americas Merino, *n* = 102; Asia, *n* = 452; Europe, *n* = 1380; Oceania, *n* = 530) and the ancestry of the 1496 Kazakhstani animals was estimated against them. In a supervised analysis, *K* is fixed by the number of reference groups, so *K* = 6 was imposed rather than selected and no cross-validation sweep was performed. This analysis used the 36,826 autosomal markers of the merged panel without LD pruning, so that it rests on the same marker set as the global PCA and neighbour-joining tree. Five independent replicates were run from different random seeds. Because the order in which ADMIXTURE returns its ancestry components is arbitrary and not guaranteed to be stable between replicates, each replicate’s components were matched to the reference groups empirically—every fixed reference animal was assigned ~1.0 in its own group’s component—and realigned before averaging. All five replicates converged to the same solution (log likelihood −192,976,468.4, agreeing across replicates to within 1 × 10^−10^ in relative terms; maximum standard deviation of any individual ancestry estimate across replicates < 0.0001).

## 3. Results

After quality control filtering, the final dataset comprised 1497 animals across three breeds—Degeres (DE, *n* = 354), Etti Merino (EM, *n* = 642), and Kazakh fat-tailed coarse-wool (KKG, *n* = 501)—sampled from seven households across Kazakhstan and genotyped at 42,279 SNPs, of which 41,292 are located on autosomes 1–26. Breed- and household-level summaries of genomic diversity are presented in Table 1 and Table 2, respectively.

All three breeds exhibited comparable expected heterozygosity (*H*_e_: DE = 0.4132, EM = 0.4174, KKG = 0.4171) and mean minor allele frequency (MAF: DE = 0.3229, EM = 0.3277, KKG = 0.3262), with fixation indices at or near zero throughout. EM and DE showed slight heterozygote excess (*F* = −0.0018 and −0.0123, respectively), while KKG was marginally positive (*F* = 0.0020). None of the three values is consistent with appreciable inbreeding. At the household level, all seven sampling units returned negative *F*, indicating a uniform mild excess of heterozygotes. The values were broadly consistent within breeds, with the most negative observed in the two smallest households—the DE Madi household (*n* = 40, *F* = −0.0290) and the KKG KH household (*n* = 23, *F* = −0.0217)—a pattern consistent with recent outcrossing or distinct founder composition rather than with population subdivision. The complete dataset with per-sample statistics is available in Supplementary Table S1.

### 3.1. Population Structure

**Figure 1:**
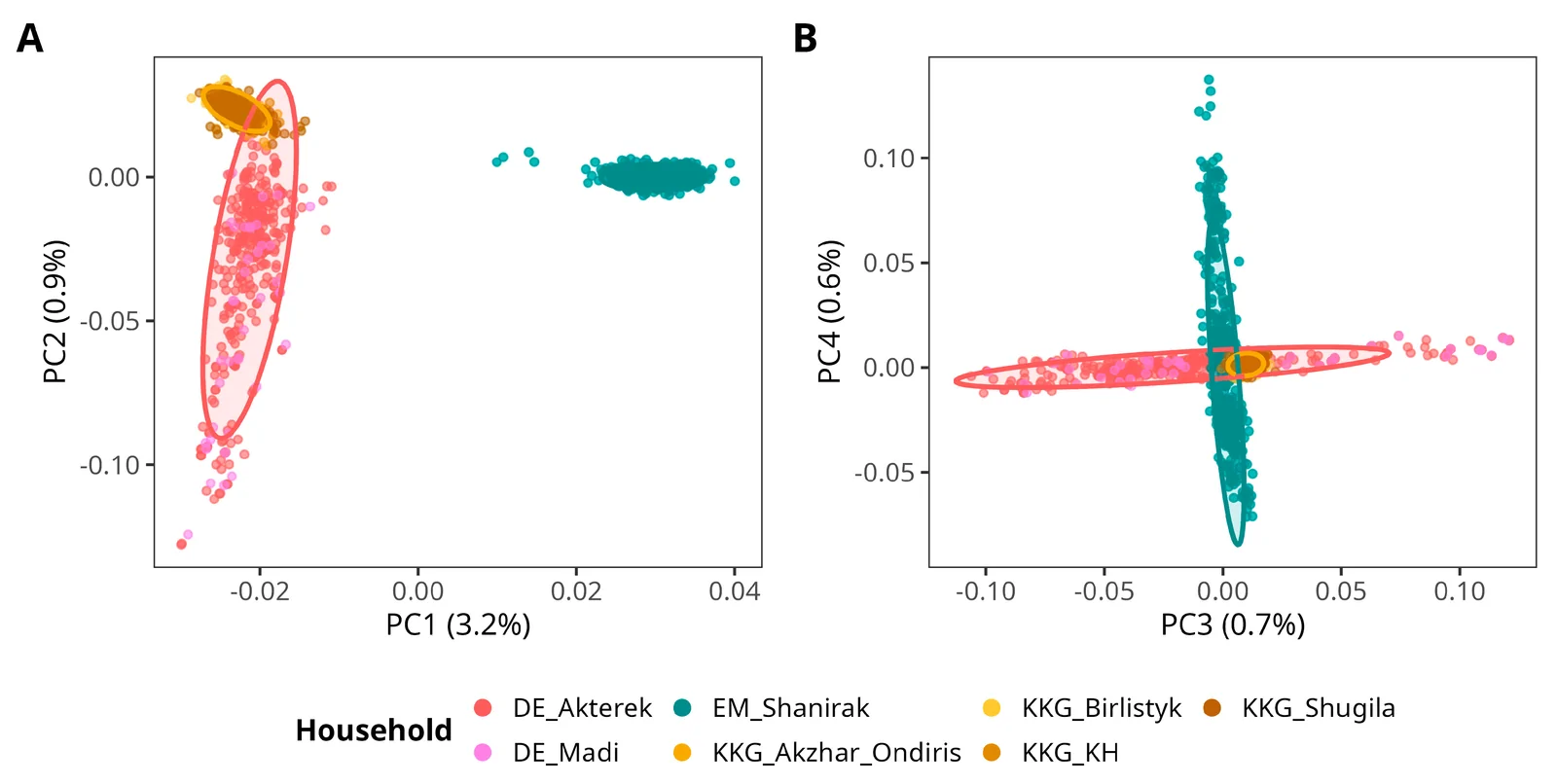
Principal component analysis of within-dataset individuals: (**A**) PC1 vs. PC2 and (**B**) PC3 vs. PC4, coloured by household.

Principal component analysis of the 1497 sheep revealed clear breed-level separation along PC1, which explained 3.2% of total variance, with PC2 accounting for 0.9% (Figure 1A). PC3 (0.7%) and PC4 (0.6%) did not reveal additional breed-level structuring beyond that captured in PC1–PC2 (Figure 1B). The absolute proportions of variance carried by the leading axes are small, as is expected for genome-wide marker data on closely related populations: the total variance is dominated by differences among individuals within breeds, and only the between-breed component—5.97% of the total by AMOVA (see below)—is available to the axes that separate breeds. PC1 and PC2 together account for 4.2%, about 70% of that available between-breed fraction, and PC1 alone separates EM from DE and KKG completely (Figure 1A).

**Figure 2:**
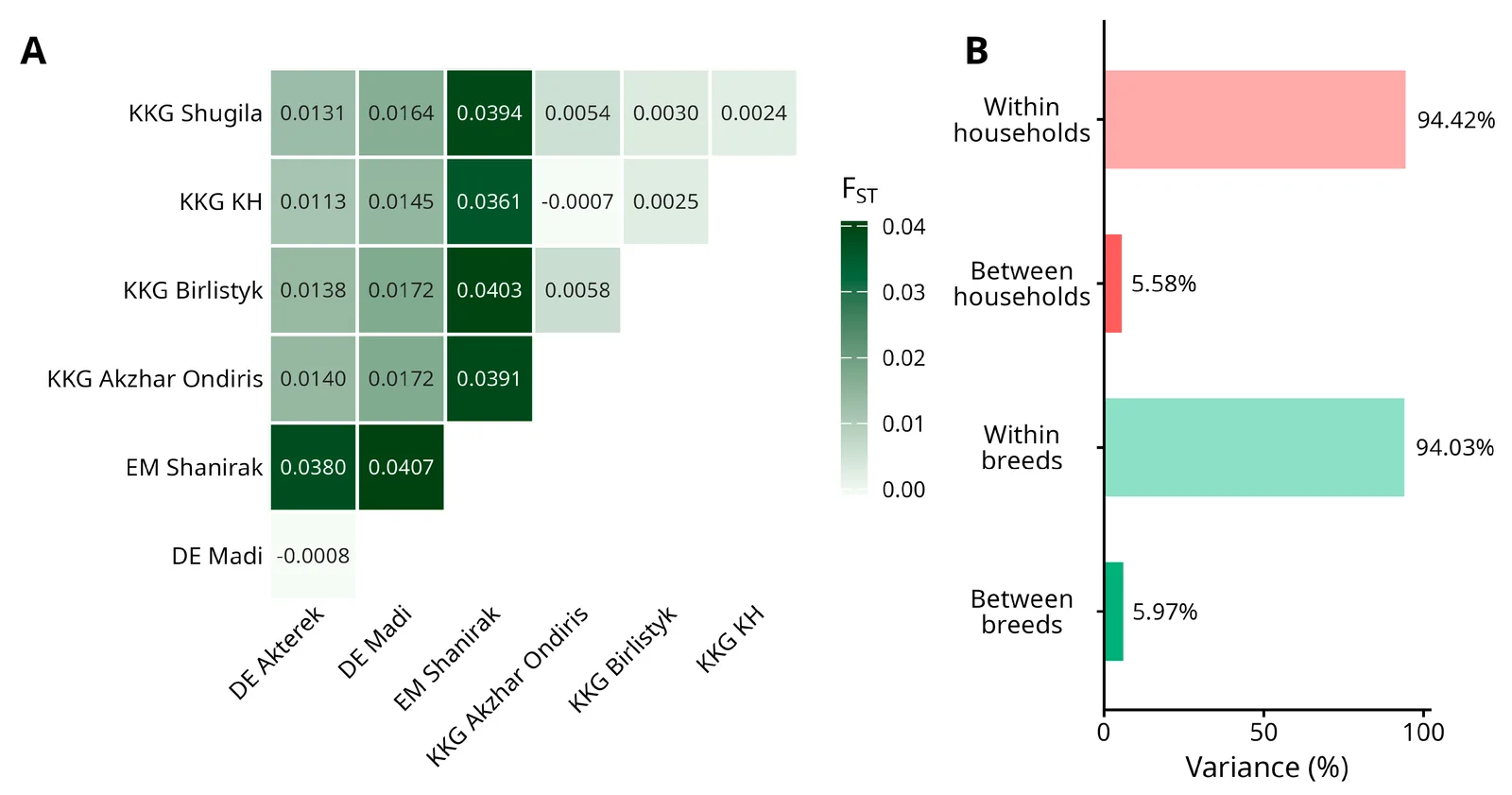
Pairwise *F*_ST_ and AMOVA results for 1497 sheep: (**A**) heatmap of pairwise *F*_ST_ values between the seven households, with colour intensity proportional to differentiation; (**B**) AMOVA-partitioned variance components at the breed and household levels.

Pairwise *F*_ST_ between households ranged from −0.0008 to 0.0407, with the largest values observed between EM_Shanirak and all DE and KKG households (Figure 2A). Within-breed household pairs showed consistently lower differentiation, particularly among KKG households (*F*_ST_ range: −0.0007 to 0.0058) and between the two DE households (*F*_ST_ = −0.0008), whereas cross-breed DE–KKG household pairs ranged from 0.0113 to 0.0172. AMOVA partitioned the majority of genomic variance within breeds (94.03%) and within households (94.42%), with 5.97% and 5.58% of variance attributable to differences between breeds (Φ_ST_ = 0.060, *p* < 0.001) and between households (Φ_ST_ = 0.056, *p* < 0.001), respectively (Figure 2B).

**Figure 3:**
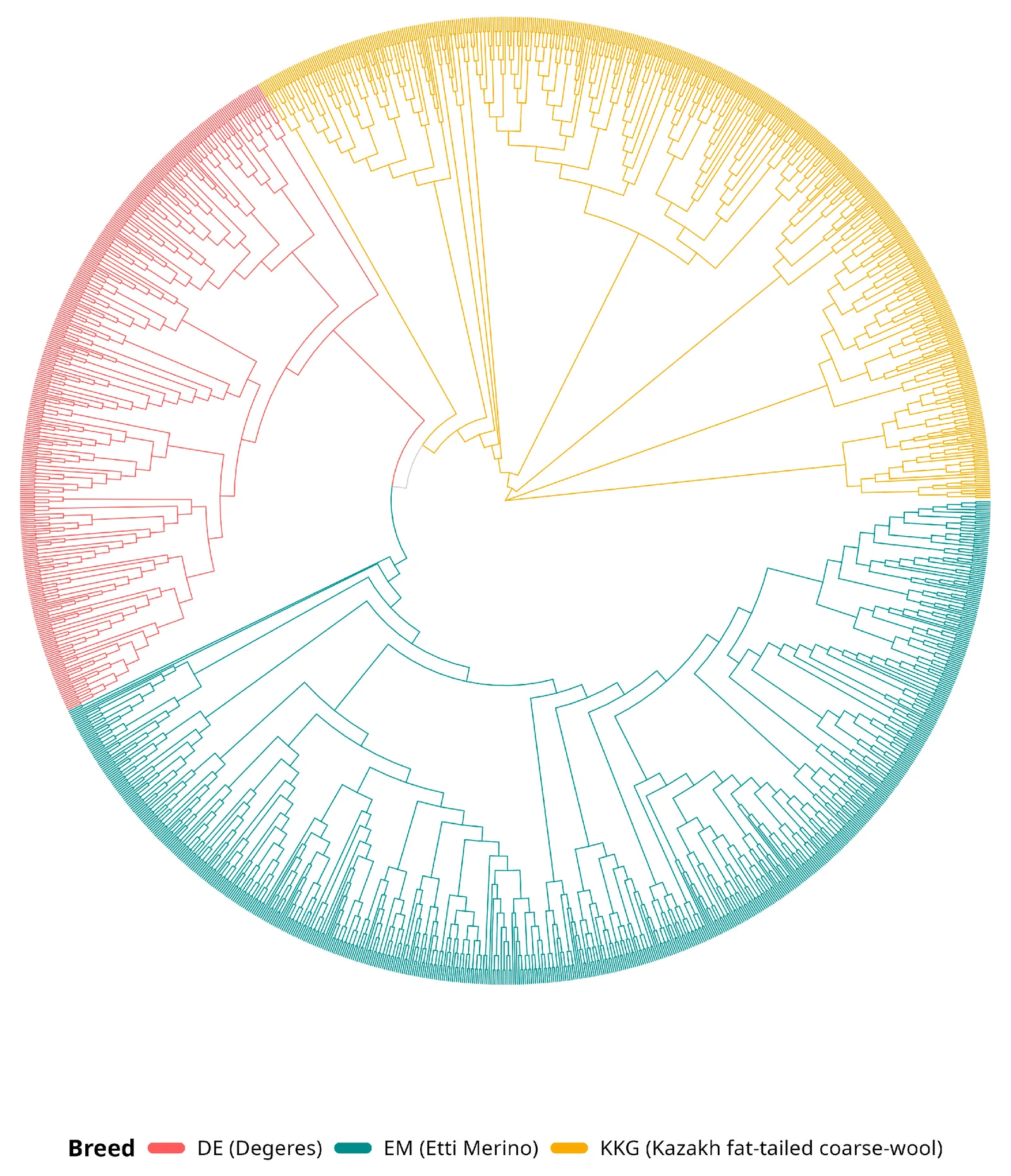
Neighbour-joining tree of 1497 sheep constructed from a genomic distance matrix, with individuals coloured by breed.

Neighbour-joining phylogenetic reconstruction of all 1497 individuals produced a topology consistent with PCA and ADMIXTURE results, with individuals grouping into three breed-concordant clades corresponding to DE, EM, and KKG (Figure 3). EM individuals formed a cohesive, well-separated clade, while DE and KKG individuals occupied adjacent clades with greater internal branching.

**Figure 4:**
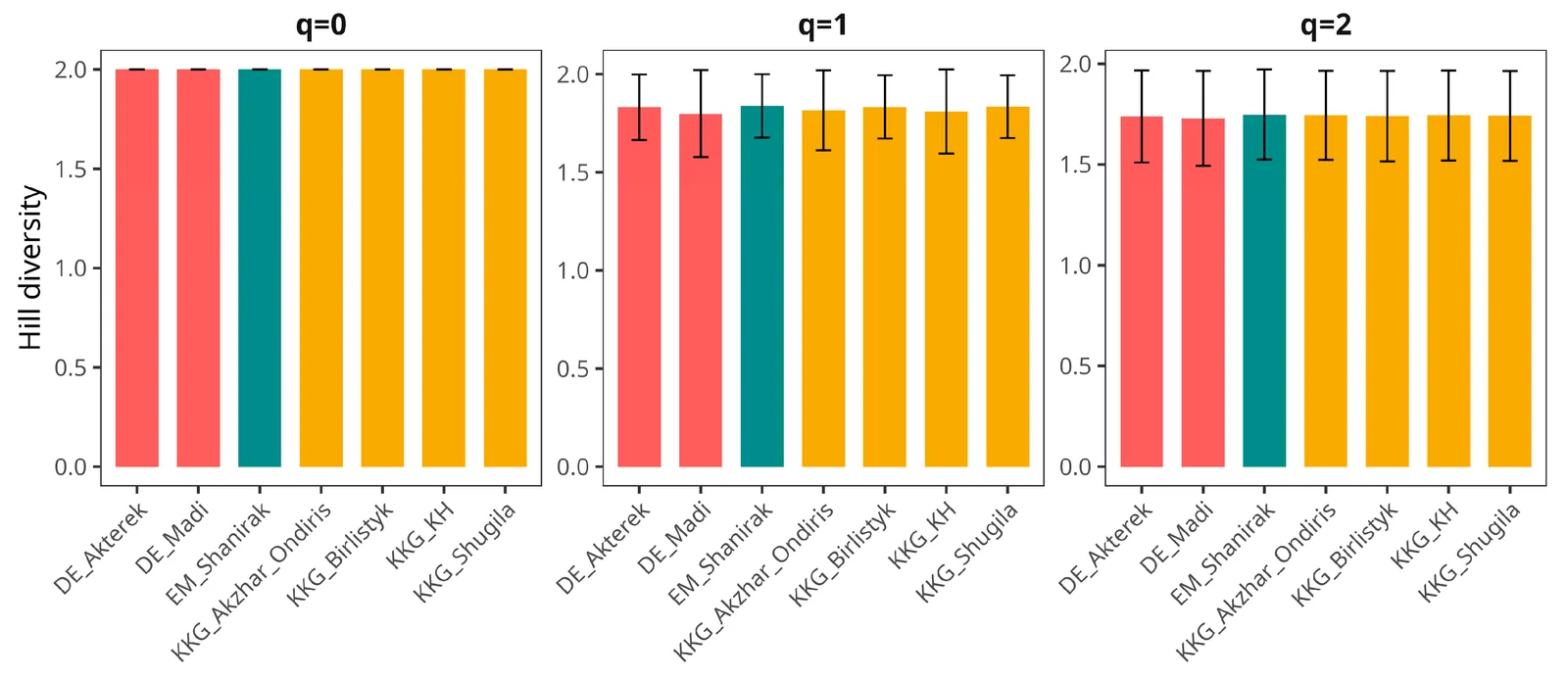
Hill-number diversity profiles across *q* = 0, *q* = 1, and *q* = 2 for all seven Kazakhstani sheep households, with error bars representing ± 1 standard error.

Hill-number diversity (*q* = 0, *q* = 1, *q* = 2) was uniformly high across all seven households, with *q* = 0 (observed richness) at approximately 2.0 for all households (Figure 4). The effective number of alleles (*q* = 1) and the inverse Simpson index (*q* = 2) were similarly consistent across households, ranging approximately 1.80–1.84 and 1.73–1.75, respectively, with overlapping error bars indicating no significant differences in diversity between households or breeds.

### 3.2. Global Population Structure

**Figure 5:**
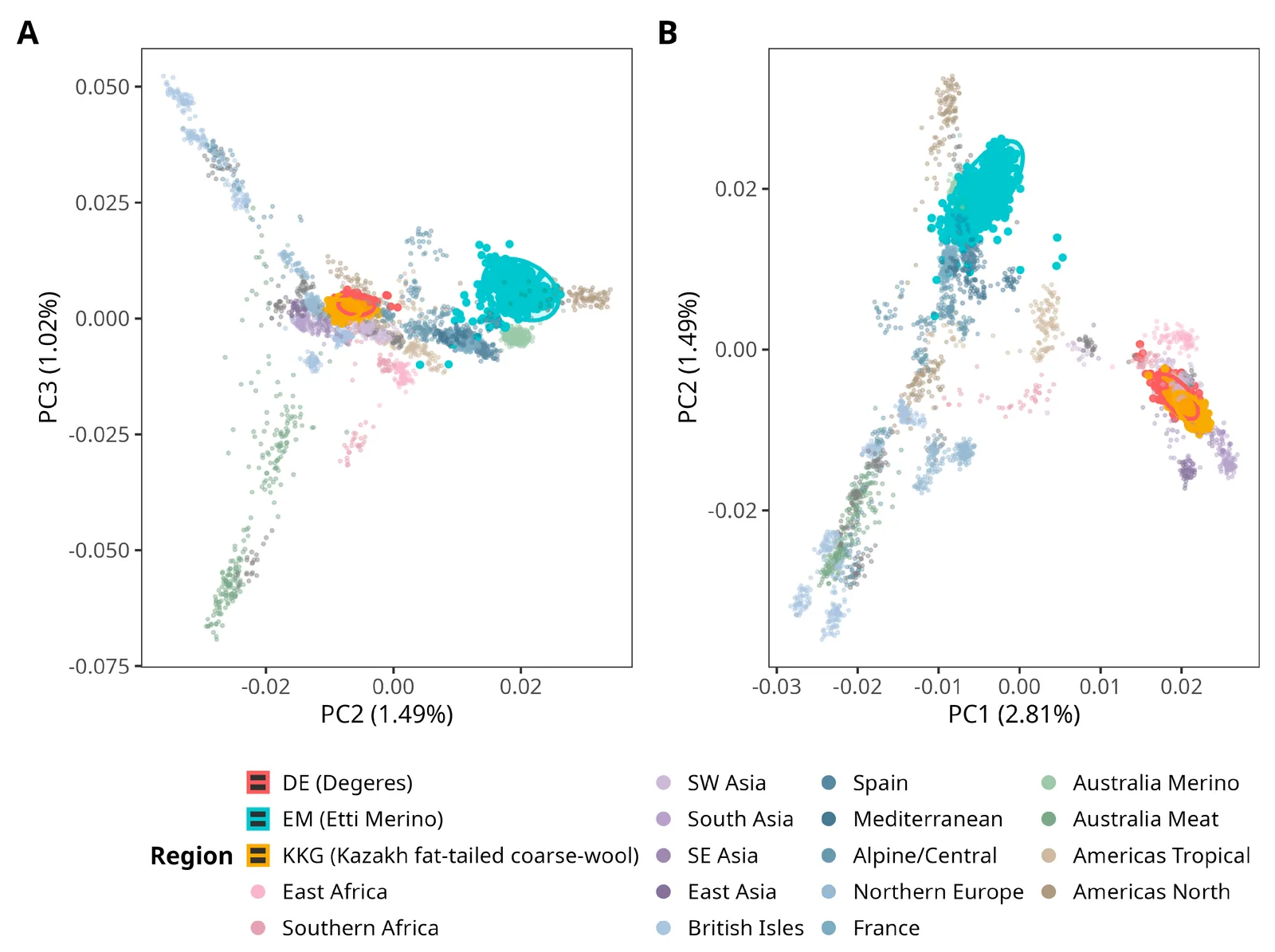
Principal component analysis of the three Kazakhstani sheep breeds projected alongside global reference populations from the Ovine SNP50 HapMap dataset: (**A**) PC3 vs. PC2 and (**B**) PC2 vs. PC1, with individuals coloured by breed (Kazakhstani) or geographic region (reference).

To contextualise the three Kazakhstani breeds within global ovine diversity, individuals were projected alongside reference populations from the Ovine SNP50 HapMap dataset using the 37,685 SNPs shared by the two array versions (4205 animals after outlier removal). In principal component analysis, PC1, PC2, and PC3 explained 2.81%, 1.49%, and 1.02% of total variance, respectively (Figure 5A,B). As in the within-dataset analysis, these proportions are low in absolute terms, and lower still here because the between-population component of variance is now distributed across the many axes required to separate 77 populations rather than three. All three Kazakhstani breeds clustered distinctly from one another and from the reference populations. EM formed a tight, well-defined cluster positioned apart from the main distribution of reference breeds, whereas DE and KKG were more proximate to one another and to the Southwest Asian and East and South Asian reference groups along PC1. Measured as the Euclidean distance between group centroids over PC1–PC5, the reference groups closest to EM were Americas North (0.018), a group dominated by the Merino-derived Rambouillet, and Australia Merino (0.030), with Southwest Asia ranked ninth (0.042). For DE and KKG, the ordering was reversed, their closest reference groups being East Asia (0.014 for both), South Asia (0.020 and 0.015), and Southwest Asia (0.026 and 0.022), with Australia Merino ranked tenth or lower (0.043).

**Figure 6:**
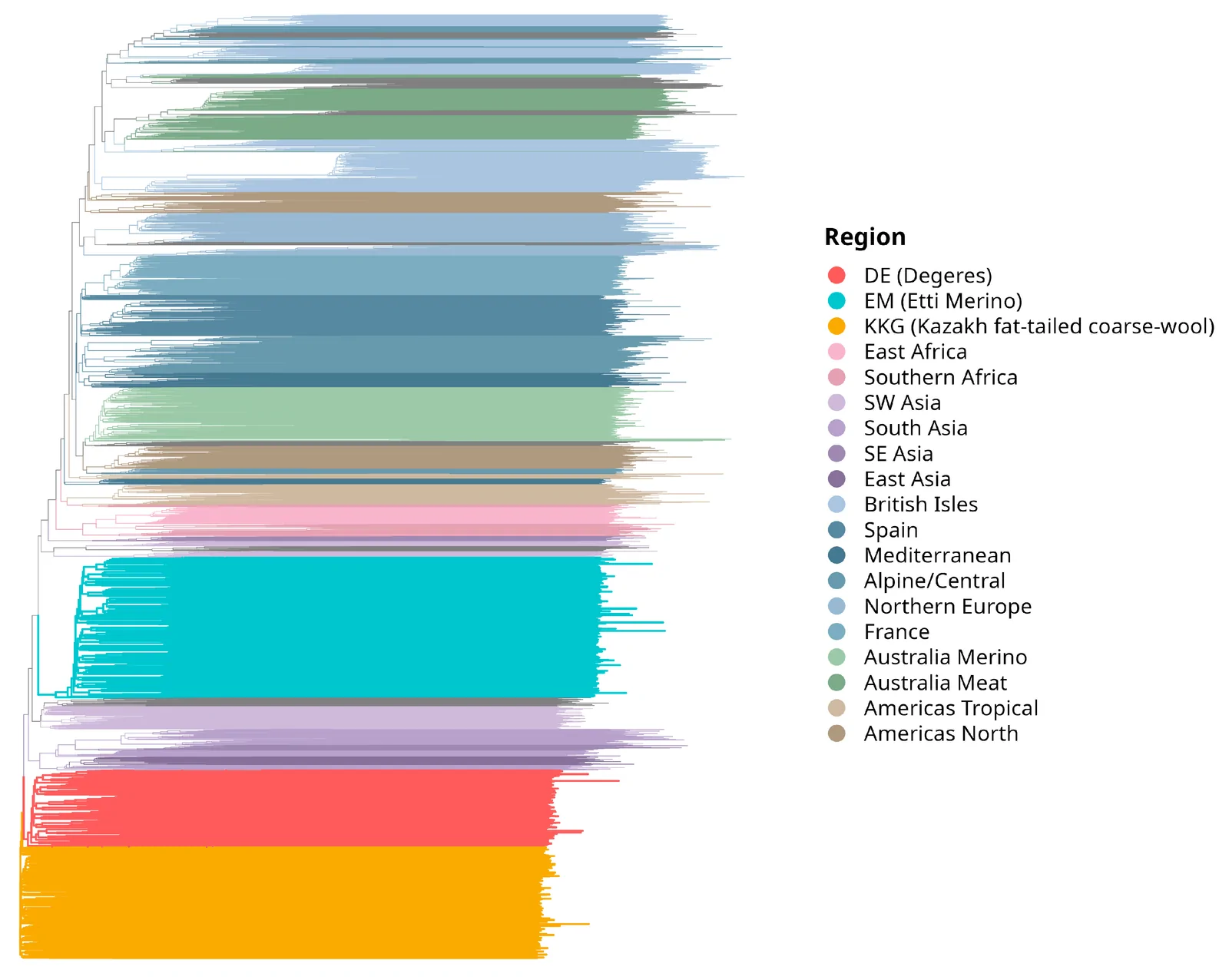
Neighbour-joining tree of the three Kazakhstani sheep breeds and global reference populations from the Ovine SNP50 HapMap dataset (4315 individuals, 36,826 autosomal SNPs), with branches coloured by breed (Kazakhstani) or geographic subregion (reference). Tip adjacency in this individual-level tree does not measure breed affinity. The quantitative affinities are given in Table 3 and Figure S1.

A neighbour-joining tree constructed from the combined Kazakhstani and global reference dataset recovered each of the three Kazakhstani breeds as a discrete, fully monophyletic clade (Figure 6). DE and KKG were reciprocal sister clades, placed together among the Southwest, East, and South Asian reference groups. EM formed a single clade containing all 642 individuals. In this individual-level topology, its immediate sister group is the clade comprising DE, KKG, and the Asian reference breeds, while the Australian Merino, Australian meat, and most European reference groups lie towards the opposite side of the tree, separated by several intervening regional clades. Tip adjacency in a tree of this size should not, however, be read as a measure of breed affinity. Neighbour-joining across 4315 individual tips is a greedy agglomerative heuristic applied to raw identity-by-state distances, which confounds between-breed divergence with within-breed heterozygosity, and for EM the resulting topology does not preserve the ordering of its own input distances: EM is closer to the Australian Merino group than to the Southwest Asian group in the input matrix (mean IBS distances 0.342 and 0.354), yet the patristic distances recovered from the tree invert this (0.363 and 0.352). The affinities of the three breeds are therefore reported below as quantities that do not depend on the drawn topology, and a neighbour-joining tree built from population-mean distances among all 77 populations is provided in Supplementary Figure S1.

Quantified as pairwise Weir and Cockerham *F*_ST_ over the 36,826 autosomal SNPs of the merged panel, the three breeds separate as their documented breeding histories predict (Table 3). EM is closest to the pooled Merino and Merino-derived reference breeds (*F*_ST_ = 0.017) and roughly 2.7 times more distant from the pooled Southwest Asian breeds (*F*_ST_ = 0.045). DE and KKG show the reciprocal pattern, closest to Southwest Asia (*F*_ST_ = 0.027 and 0.020) and considerably more distant from the Merino group (*F*_ST_ = 0.052 for both). EM is also more distant from DE and from KKG (*F*_ST_ = 0.041 for both) than it is from the Merino reference breeds. Ranked across all 74 reference breeds, the eight nearest to EM are Rasa Aragonesa, Rambouillet, Australian Poll Merino, Australian Merino, Australian Industry Merino, Ojalada, Chinese Merino, and Castellana—Spanish and Merino-derived populations exclusively—whereas the nearest to DE and to KKG are the Southwest Asian Qezel, Moghani, and Afshari. The breed-level neighbour-joining tree (Figure S1) recovers the same structure topologically: EM is placed as sister to Chinese Merino and Rambouillet within a clade that also contains the four Australian Merino breeds, Merinolandschaf and the Spanish breeds, and DE and KKG are reciprocal sisters embedded among the Southwest and South Asian breeds.

### 3.3. Ancestry Estimation

**Figure 7:**
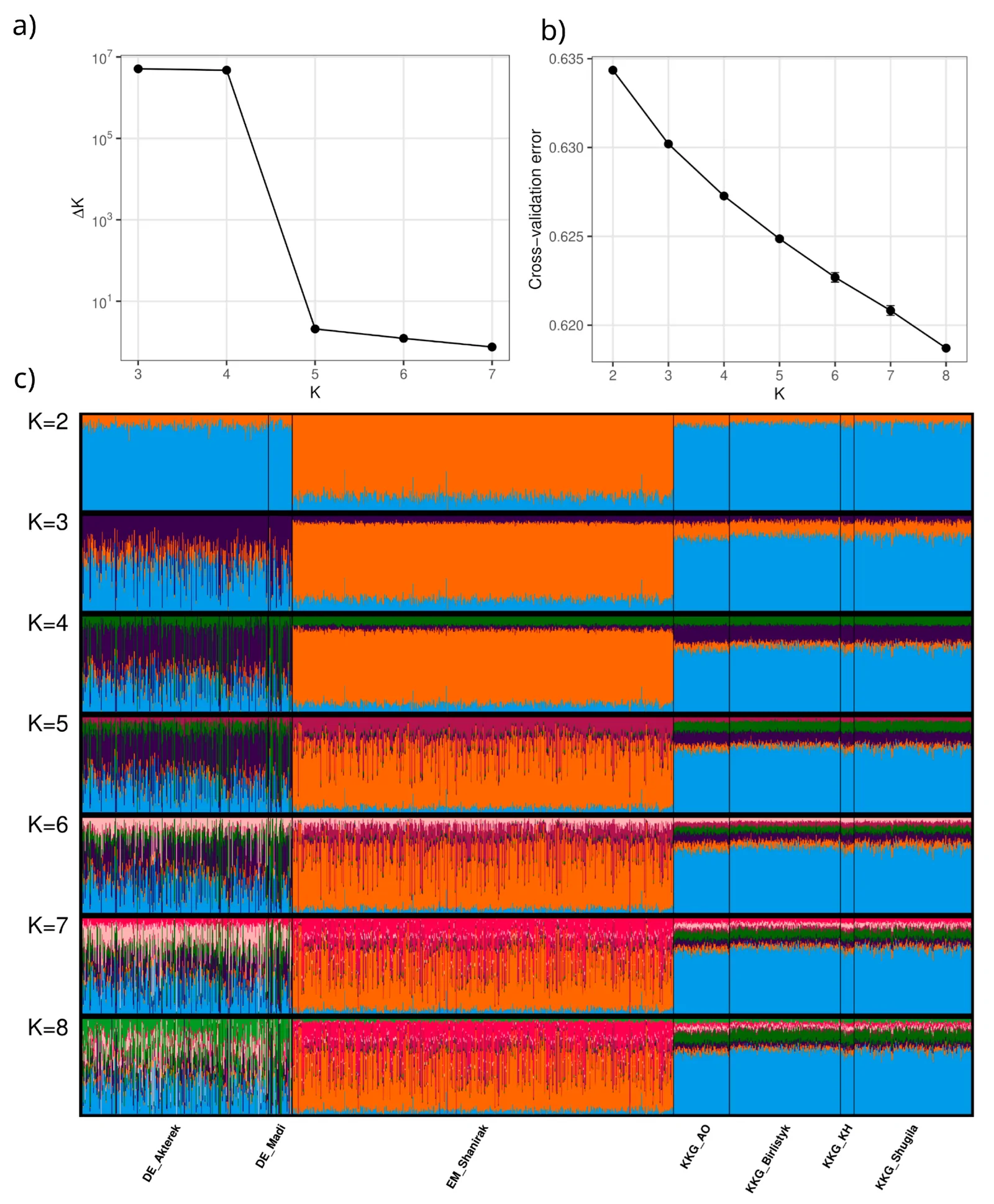
Admixture analysis results: (**a**) delta *K* (Δ*K*) statistics across *K* = 2 to *K* = 8 ancestry components; (**b**) cross-validation error across *K* = 2 to *K* = 8 ancestry components; (**c**) ADMIXTURE bar plots for *K* = 2 through *K* = 8, with individuals grouped by household.

Ancestry proportion estimation across *K* = 2 to *K* = 8 ancestry components is summarised in Figure 7. Δ*K* peaked at *K* = 3 (Figure 7a), identifying it as the most supported number of ancestry components. Cross-validation error decreased monotonically from *K* = 2 to *K* = 8, without a distinct minimum (Figure 7b). At *K* = 3, EM and KKG individuals were each assigned predominantly to a single breed-concordant ancestry component (Figure 7c). DE individuals, by contrast, showed substantial shared ancestry with the KKG-associated component across the breed as a whole rather than in a minority of individuals, particularly in the Akterek household, consistent with DE’s documented formation from Kazakh fat-tailed stock crossed with introduced breeds. From *K* = 4 onward, progressive fragmentation of ancestry components was observed within breeds with no emergence of new discrete clusters, further supporting *K* = 3 as the optimal and biologically meaningful solution (Figure 7).

**Figure 8:**
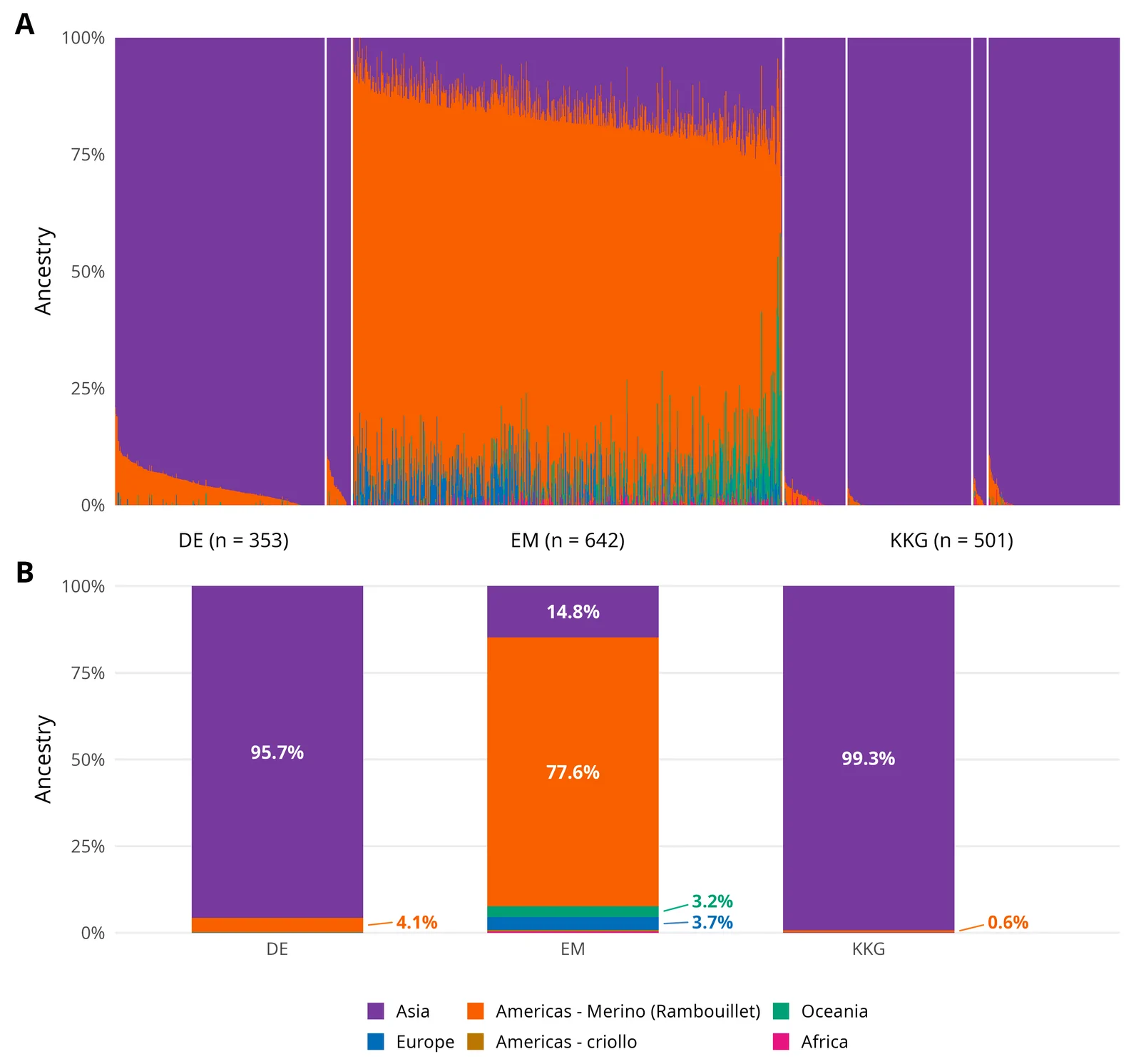
Supervised ADMIXTURE ancestry of the three Kazakhstani breeds against the global reference panel (*K* = 6, 36,826 autosomal SNPs, mean of five replicates). The 2819 reference animals were fixed to their continental groups and are not plotted, since they are assigned to their own component by construction. Only the 1496 estimated Kazakhstani animals are shown. (**A**) Per-individual ancestry proportions ordered by breed and household, with white rules at household boundaries; (**B**) breed means.

To estimate ancestry against the global reference panel rather than within the three breeds alone, a supervised ADMIXTURE analysis was run at *K* = 6 with the 2819 reference animals fixed to their continental groups (Figure 8). The three breeds separated as the *F*_ST_ values in Table 3 and the population-mean tree in Figure S1 predict. EM carried a majority Merino-derived ancestry: 77.6% was assigned to the Americas Merino component, which is anchored by Rambouillet, the only dedicated Merino-derived reference breed in the panel, with a further 3.2% Oceanian and 3.7% European ancestry, while Asian ancestry accounted for only 14.8%. DE and KKG showed the reciprocal composition, at 95.8% and 99.3% Asian ancestry, respectively, with the Merino-derived component reduced to 4.1% in DE and 0.6% in KKG. The contrast between EM and the two indigenous-ancestry breeds is therefore recovered by a third, independent method against the same reference panel used for the global PCA and phylogeny. Two qualifications apply to these proportions. A supervised analysis constrains every estimated animal to be a mixture of the reference groups supplied, so the values are ancestry relative to these six groups rather than absolute ancestry, and the Oceanian reference group pools Australian Merino with Australian meat breeds, so it is not a clean Merino proxy, which is why EM’s Merino signal loads chiefly on the Rambouillet-anchored component. Per-household ancestry proportions are given in Supplementary Table S4.

## 4. Discussion

This study provides the first genome-wide SNP characterisation to include Degeres (DE), Etti Merino (EM), and Kazakh fat-tailed coarse-wool (KKG) sheep simultaneously, completing a portrait that prior work had assembled only in part—from early microsatellite surveys of the Kazakh portfolio [7,21,24] to recent SNP-array studies [22,23]. The most informative result is not the within-dataset separation of the three breeds, which principal component analysis, neighbour-joining phylogeny, and ADMIXTURE all recover concordantly at *K* = 3, but the partition that emerges when all three are projected against the global reference panel. EM is genetically closest to Merino and Merino-derived populations, whereas DE and KKG are closest to Southwest, East, and South Asian breeds, each being more distant from the other’s nearest reference group than from its own (Table 3, Figure 5 and Figure S1). This biogeographic split has a deep historical scaffold. Recent archaeogenomic work places sheep domestication near central Anatolia and documents a major Bronze Age influx of Western steppe-related ancestry into domestic sheep that parallels contemporaneous human migrations [2,3], and whole-genome resequencing has traced the introgression corridors linking the Eurasian steppe to the Iranian plateau [4]. Microsatellite and SNP surveys across northern Eurasia and East Asia have likewise resolved a distinct fat-tailed and a composite cluster separable from fine-wool types [15,17,36]. The placement of DE and KKG among Asian breeds is consistent with the steppe ancestry shared across Central Asia. EM’s projection away from its geographic neighbours, despite its development and ongoing production on Kazakhstani soil, instead reflects the persistence of a Merino genomic signature: once established through sustained introgression of Merino-type rams—in EM’s case, German Mutton Merino (Merinofleischschaf) rams crossed onto Kazakh fine-wool stock to found the breed [8]—this signature is retained across generations of local adaptation and remains the dominant component of ancestry irrespective of geographic context.

Merino and Merino-derived populations worldwide share a distinct ancestry component traceable to an Iberian origin, with haplotype sharing detectable preferentially among breeds of Merino descent [37,38], and the same signature is recoverable through the wool-selection architecture shared across Spanish, Australian, and Chinese Merino lines [39]. This persistence is now documented across three continents independently. Among the Merino and Merino-derived breeds that descend from the Spanish Merino founder population, among-breed variance accounts for only about 5% of the total despite centuries of closed-line breeding [19], a partition comparable to the 5.97% we recovered between our breeds. In South Africa, the Merino-derived Dohne Merino, Meatmaster, and Afrino each align with their Merino ancestors along a principal axis and retain >80% within-population variance [20], and whole-genome scans recover Merino-type haplotypes shared among Australian, Chinese, and South African Merino at growth loci including *GHR*, *LCORL*, and *NCAPG* [40]. Against this backdrop, EM’s divergence from the indigenous breeds is quantitatively modest: the maximum pairwise *F*_ST_ between EM and any DE or KKG household was 0.041, a value that falls within the range documented among Merino bloodlines and strains themselves rather than between Merino and unrelated stock [41]. The direct implication for breed management is that EM, DE, and KKG represent three distinct gene pools, and managing EM within a shared selection framework risks treating samples from three ancestries as one, confounding precisely the traits that justify maintaining the breeds separately.

Against EM’s clean and cohesive ancestry profile, DE carries the signature of a composite breed whose consolidation is incomplete. Three lines of evidence converge. First, every DE sampling unit returned a negative fixation index, with the breed as a whole the most heterozygote-excess of the three (*F* = −0.0123, versus −0.0018 for EM and +0.0020 for KKG) and the smaller Madi household the most negative of all seven households (*F* = −0.0290), the excess of heterozygotes expected when a breed is still being assembled by crossing not-yet-fixed founder lines rather than reproducing as a single closed pool. Second, DE individuals as a whole, not merely a subset, carried substantial shared ancestry with the KKG-associated component at *K* = 3, a pattern essentially absent from EM and much reduced in KKG. Against the global reference panel, DE also retained a residual 4.1% Merino-derived component (Figure 8) against 0.6% in KKG, which is consistent with the Merino-derived Precoce used in the breed’s formation [5,6]. Third, the two DE households differed in their internal profiles, with the smaller Madi household diverging from Akterek. This is the canonical signature of an unconsolidated composite, and it now has close genomic analogues. The US Polypay shows clear residual substructure and differentiation from its foundation flock [42]. The composite US Katahdin retains flock-level genetic connectedness traceable to animal sharing among breeders [43]. The German White-Headed Mutton, improved through crossing, still carries detectable introgressed ancestry from the breeds used in its improvement [44], and admixed South African composites likewise retain quantifiable founder-breed proportions long after formation [45]. The inter-household *F*_ST_ among our DE and KKG flocks (−0.0008 to 0.0172) sits well below the 0.034–0.094 reported for the informally managed Santa Inês composite [46] and the inter-flock differentiation seen in US Suffolk [47], indicating that DE has not yet fragmented to that degree. The heterozygote excess and partial admixture we observe are most parsimoniously read as the early signal of a composite still being consolidated, consistent with DE’s documented breeding history, a multi-breed composite (Kazakh fat-tailed, Shropshire, and Precoce) whose second intra-breed type was tested only in 2009 and is still propagated by ‘breeding in itself’ of successive hybrid generations rather than as a closed pool [5,6]—with the synthetic origin and low interbreed differentiation reported for Kazakh breeds from microsatellites [7], and with the inconsistent selection practices documented for Kazakhstani semi-coarse-wool stock [22]. Because our breeds differ substantially in sample size and in the number of households sampled—EM is represented by a single household versus two for DE and four for KKG—this interpretation is offered with the caveat that imbalanced sampling can itself bias model-based clustering [48] and that EM’s apparent homogeneity has not been tested for inter-household variation in the way DE’s and KKG’s have. The corroborating *F*_ST_ and fixation-index evidence, however, does not depend on the ADMIXTURE solution alone.

Allelic diversity within all three breeds was uniformly high and exceeded most published regional comparators. Observed heterozygosity ranged from 0.4157 (KKG) to 0.4183 (DE) and expected heterozygosity from 0.4132 (DE) to 0.4174 (EM), exceeding the *H*_o_ range of 0.354–0.395 across 25 Russian breeds [18] and the values reported for indigenous Iranian breeds [16,49]. Mean minor allele frequency ranged from 0.323 to 0.328, likewise exceeding that of five Chinese indigenous breeds [50], with fixation indices at or near zero throughout. The near-zero to slightly negative *F* values match the pattern reported across indigenous Chinese stock [50], and the Hill-number profiles, flat across *q* = 0, *q* = 1, and *q* = 2 with overlapping error bars across all seven households, confirm even allele-frequency distributions without dominance by a few high-frequency haplotypes [51], inconsistent with the bottlenecks reported for endangered breeds in which genomic Ne collapses to low double digits [52]. AMOVA partitioned 94.03% of variance within breeds (Φ_ST_ = 0.060, *p* < 0.001) and 94.42% within households (Φ_ST_ = 0.056, *p* < 0.001), consistent with the shallow breed-formation history and pervasive within-breed diversity characteristic of sheep globally [1]. Far from indicating weak breed identity, this partition is the expected genome-wide norm: a survey of more than 35,000 breed pairs found that between-breed *F*_ST_ values are typically modest and that the common threshold of *F*_ST_ > 0.1 is not a reliable criterion for breed distinctiveness [53]. The shallow among-group differentiation we report therefore reflects the recent, reticulate history of breed formation rather than any deficiency in diversity. The same shallow differentiation accounts for the low proportion of variance carried by the leading principal components, and is a direct consequence of it rather than an independent limitation of the analysis. In a principal component analysis of genome-wide genotypes, the total variance is overwhelmingly made up of differences among individuals within populations: only the between-population fraction—here 5.97% by AMOVA—can be expressed on the axes that separate the groups. PC1 and PC2 of the within-dataset analysis together account for 4.2%, about 70% of that available fraction, and in the global analysis the corresponding fraction is divided among the many more axes needed to separate 77 populations, which is why the individual percentages there are smaller still. Low absolute percentages of this kind are the expected outcome for closely related livestock breeds genotyped at genome-wide density and do not indicate weak or unreliable structure. What matters for the inference is whether the leading axes resolve the groups, which here they do completely. This genomic baseline establishes the conditions for the logical next step: selection-signature mapping against three contrasting ancestry backgrounds. For the fat-tailed KKG in particular, the candidate architecture of tail-fat adipogenesis is now well defined, with *BMP2*, *PDGFD*, and *VRTN* repeatedly nominated across Chinese fat-tailed and fat-rumped breeds [54,55], the regulatory and metabolic context increasingly resolved through multi-omic work [56], ROH-island selection signatures already reported in KKG itself [57], and causal *PDGFD* and *BMP2* variants, functionally validated in vitro, identified in fat-tailed sheep including the Kazakh clade [58]; the uniform diversity documented here makes analogous mapping directly tractable in DE and EM as well.

Taken together, these findings bear on how sheep breeding in Central Asia has actually proceeded and on what that means for regional agriculture. The three breeds represent three distinct routes to a productive population: an indigenous breed shaped predominantly by natural selection under nomadic pastoralism (KKG), a recent composite assembled by planned crossing and still consolidating (DE), and a specialist of introduced ancestry maintained for a commodity trait under local conditions (EM). The genomic data show that all three have been arrived at without measurable loss of diversity, which is not a trivial outcome: comparable Merino-derived and composite breeds elsewhere have lost variability during their formation [20,42,44], whereas the Kazakhstani populations retain heterozygosity at or above the levels reported for indigenous Russian, Iranian, and Chinese stock [16,18,50]. The significance for agriculture is twofold. First, the country’s mutton and wool production rests on a genetically broad base that can absorb sustained selection without immediate inbreeding risk. Second, the three breeds are complementary rather than redundant: they occupy different positions on both the coarse-wool to fine-wool axis and the indigenous to introduced-ancestry axis, and each therefore carries allelic variation the other two do not, which is precisely the configuration that makes a national flock resilient to changing market and climate demands.

## 5. Conclusions

This study delivers the first genome-wide characterisation of DE, EM, and KKG together, resolving three findings with direct consequences for breed management. EM retains a Merino genomic signature strong enough that it is genetically closer to Merino and Merino-derived reference breeds (pooled *F*_ST_ = 0.017) than to the Kazakh breeds farmed alongside it in the merged global panel (*F*_ST_ = 0.041) or to its Southwest Asian geographic neighbours (*F*_ST_ = 0.045), despite over a decade of local breeding. DE carries converging signatures of an incompletely consolidated composite (heterozygote excess, partial non-DE ancestry at *K* = 3, inter-household divergence), consistent with its documented multi-breed origin. Finally, all three breeds retain uniformly high within-breed diversity (*H*_e_ = 0.413–0.417), with the great majority of variance partitioned within rather than between breeds (Φ_ST_ = 0.060) or households (Φ_ST_ = 0.056). None of the three shows evidence of inbreeding or diversity loss.

For the structure of the Kazakhstani sheep population, these results carry four practical implications. First, the three breeds should be recorded and selected as three separate populations: treating EM as interchangeable with the indigenous fat-tailed breeds, or DE as a fully closed population, would obscure the ancestry differences documented here and confound the traits that justify keeping the breeds apart. Second, because EM’s ancestry is Merino rather than Central Asian, the selection indices, reference populations, and genomic prediction equations developed for Merino-type sheep elsewhere are the appropriate starting point, whereas DE and KKG will require a reference base built on Central Asian fat-tailed stock. Pooling the three for genomic evaluation would degrade prediction in all three. Third, because roughly 94% of the genetic variance lies within rather than between breeds, genetic progress in this system is available through within-breed selection rather than through further crossbreeding, and the uniformly high heterozygosity and near-zero fixation indices indicate that selection intensity can be raised without immediate inbreeding risk, provided effective population size is monitored. Fourth, DE is where consolidation effort is best directed: its residual KKG-associated ancestry and inter-household divergence identify a composite still being assembled, and a defined nucleus flock with recorded matings would fix the type more rapidly than the current practice of breeding in itself. The uniform diversity baseline established here also makes all three breeds directly tractable for selection-signature mapping, particularly around the fat-tail adipogenesis loci already characterised in KKG’s relatives, providing a genomic foundation for both breed conservation and applied selection in Kazakhstani sheep.

## Figures and Tables

**Table 1:** Breed-level summary statistics of genomic diversity for three sheep breeds.

Breed	*N*	*H*_o_	*H*_e_	*F*	MAF
Degeres	354	0.4183	0.4132	−0.0123	0.3229
Etti Merino	642	0.4181	0.4174	−0.0018	0.3277
Kazakh fat-tailed coarse-wool	501	0.4157	0.4171	0.0020	0.3262

*N*, number of animals; *H*_o_, observed heterozygosity, averaged over markers (PLINK --hardy); *H*_e_, expected heterozygosity under Hardy–Weinberg equilibrium, averaged over markers (PLINK --hardy); *F*, method-of-moments inbreeding coefficient per animal, *F* = (O(HOM) − E(HOM))/(N(NM) − E(HOM)), averaged over animals (PLINK --het), where negative values denote an excess of observed heterozygotes relative to Hardy–Weinberg expectation; MAF, minor allele frequency, averaged over markers (PLINK --freq). *H*_o_, *H*_e_, and MAF were computed on the full 42,279-SNP panel and *F* on the 41,296 autosomal and unplaced markers scanned by PLINK --het, with allele frequencies estimated within the breed shown.

**Table 2:** Household-level summary statistics of genomic diversity across seven sampling units within three sheep breeds.

Breed	Household	*N*	*H*_o_	*H*_e_	*F*	MAF
DE	Akterek	314	0.4183	0.4135	−0.0117	0.3232
DE	Madi	40	0.4217	0.4094	−0.0290	0.3191
EM	Shanirak	642	0.4181	0.4174	−0.0018	0.3277
KKG	Akzhar Ondiris	94	0.4179	0.4163	−0.0065	0.3256
KKG	Birlistyk	187	0.4161	0.4147	−0.0031	0.3240
KKG	KH	23	0.4213	0.4158	−0.0217	0.3254
KKG	Shugila	197	0.4151	0.4152	−0.0012	0.3243

*N*, number of animals; *H*_o_, observed heterozygosity, averaged over markers (PLINK --hardy); *H*_e_, expected heterozygosity under Hardy–Weinberg equilibrium, averaged over markers (PLINK --hardy); *F*, method-of-moments inbreeding coefficient per animal, *F* = (O(HOM) − E(HOM))/(N(NM) − E(HOM)), averaged over animals (PLINK --het), where negative values denote an excess of observed heterozygotes relative to Hardy–Weinberg expectation; MAF, minor allele frequency, averaged over markers (PLINK --freq). *H*_o_, *H*_e_, and MAF were computed on the full 42,279-SNP panel and *F* on the 41,296 autosomal and unplaced markers scanned by PLINK --het, with allele frequencies estimated within the household shown.

**Table 3:** Pairwise Weir and Cockerham *F*_ST_ between each Kazakhstani breed and selected reference breeds and pooled reference groups of the OvineSNP50 HapMap panel.

Reference Group or Breed	*n*	EM	DE	KKG
Merino and Merino-derived (pooled) ^a^	385	0.017	0.052	0.052
Rambouillet	102	0.029	0.075	0.076
Australian Poll Merino	98	0.030	0.059	0.058
Australian Merino	50	0.031	0.060	0.059
Australian Industry Merino	88	0.032	0.061	0.060
Chinese Merino	23	0.033	0.071	0.071
Merinolandschaf	24	0.044	0.073	0.072
Spanish breeds: Rasa Aragonesa	22	0.023	0.047	0.045
Ojalada	24	0.032	0.055	0.054
Castellana	23	0.035	0.057	0.055
Southwest Asian (pooled) ^b^	219	0.045	0.027	0.020
Qezel	35	0.050	0.027	0.019
Moghani	34	0.054	0.032	0.024
Afshari	37	0.071	0.050	0.043
Kazakhstani: DE (Degeres)	353	0.041	-	0.013
Kazakhstani: KKG (Kazakh fat-tailed coarse-wool)	501	0.041	0.013	-

*F*_ST_ values are Weir and Cockerham (1984) [28] estimates computed on the 36,826 autosomal SNPs of the merged 37,685-SNP dataset; *n*, number of reference animals. ^a^ Rambouillet, Australian Merino, Australian Poll Merino, Australian Industry Merino, Chinese Merino, and Merinolandschaf pooled; Macarthur Merino (*n* = 10), a small closed historical flock strongly drifted from every other population in the panel (*F*_ST_ > 0.17 against all three study breeds), is excluded. ^b^ The eight breeds assigned to the Southwest Asian subregion in Table S2, pooled. DE is represented by 353 animals in the merged dataset, one individual having been lost at the merge step.

## Data Availability

The data are contained within the article. The genotype data (PLINK binary format) generated for the Kazakh fat-tailed coarse-wool, Degeres, and Etti Merino breeds in this study are deposited in the Zenodo repository at https://doi.org/10.5281/zenodo.21101034.

## Supplementary Materials

The following supporting information can be downloaded at https://www.mdpi.com/article/10.3390/ani16172754/s1. Table S1: Per-sample genomic diversity statistics for all 1497 sheep in the study dataset (Degeres, DE; Etti Merino, EM; Kazakh fat-tailed coarse-wool, KKG). Table S2: Composition of the ISGC OvineSNP50 HapMap reference panel used for the global PCA (Figure 5) and the neighbour-joining trees (Figure 6 and Figure S1): per-breed sample sizes and geographic group assignments for all 74 reference breeds. Table S3: Nei-corrected net genomic distances between population means for all 77 populations (74 ISGC OvineSNP50 HapMap reference breeds and the three Kazakhstani study breeds). Table S4: Supervised ADMIXTURE ancestry proportions (%) of the three Kazakhstani sheep breeds against the global reference panel, by sampling household. Figure S1: Neighbour-joining tree of the 77 populations constructed from population-mean genomic distances, with tips coloured by continental group.

## Abbreviations

The following abbreviations are used in this manuscript:

AMOVA	analysis of molecular variance
DE	Degeres
EM	Etti Merino
*F*	inbreeding coefficient (method-of-moments estimate)
*F*_ST_	fixation index, a measure of genetic differentiation between populations
*H*_e_	expected heterozygosity
*H*_o_	observed heterozygosity
IBS	identity by state
ISGC	International Sheep Genomics Consortium
KKG	Kazakh fat-tailed coarse-wool
LD	linkage disequilibrium
MAF	minor allele frequency
*N*, *n*	number of animals
NJ	neighbour-joining
PCA	principal component analysis
QC	quality control
SNP	single-nucleotide polymorphism

## References

[1] Kijas J.W., Lenstra J.A., Hayes B., Boitard S., Porto Neto L.R., San Cristobal M., Servin B., McCulloch R., Whan V., Gietzen K. (2012). Genome-wide analysis of the world’s sheep breeds reveals high levels of historic mixture and strong recent selection. PLoS Biol..

[2] Kaptan D., Atağ G., Vural K.B., Morell Miranda P., Akbaba A., Yüncü E., Buluktaev A., Abazari M.F., Yorulmaz S., Kazancı D.D. (2024). The population history of domestic sheep revealed by paleogenomes. Mol. Biol. Evol..

[3] Daly K.G., Mullin V.E., Hare A.J., Halpin Á., Mattiangeli V., Teasdale M.D., Rossi C., Geiger S., Krebs S., Medugorac I. (2025). Ancient genomics and the origin, dispersal, and development of domestic sheep. Science.

[4] Lv F.H., Cao Y.H., Liu G.J., Luo L.Y., Lu R., Liu M.J., Li W.R., Zhou P., Wang X.H., Shen M. (2022). Whole-genome resequencing of worldwide wild and domestic sheep elucidates genetic diversity, introgression, and agronomically important loci. Mol. Biol. Evol..

[5] Adylkanova S.R., Sadykulov T., Kim G.L., Koishibayev A.M., Dolgopolova S.Y. (2018). The biological growth and development of lambs degeres fat-tailed sheep breed. EurAsian J. BioSci..

[6] Adylkanova S.R., Sadykulov T., Kim G.L., Koishibayev A.M., Baimazhi E.B., Dolgopolova S.Y. (2019). Transamination enzymes and their role in the selection of degeres sheep. EurAsian J. BioSci..

[7] Ozerov M.Y., Marzanov N.S., Tapio M., Burabaev A.A., Marzanova L.K., Amerkhanov K.A., Kantanen Y. (2008). Genetic features of Kazakh sheep breeds according to microsatellites. Russ. Agric. Sci..

[8] Torekhanov A.A., Zhumadillayev N.K. (2021). Etti Merinos-Kazakhstani export-oriented meat bread. Rep. Natl. Acad. Sci. Repub. Kazakhstan.

[9] Zhumadillayev N.K., Yuldashbaev Y.A., Karynbaev A.K. (2020). Effect of hornless on the productivity of Etti Merino sheep. Agrar. Sci..

[10] Basitov K.T., Yuldashbayev Y.A., Prmanshaev M. (2023). Milk productivity of meat breeds of sheep of different origin in the south-east of Kazakhstan. Sheep Goats Wool Bus..

[11] Khamzin K., Omarova K., Smagulov D., Orkara S., Beken A., Kozhayeva A., Khamzina A., Yesengaliyev K., Sharapatov T. (2026). Kazakh sheep review: History, breeds, genetic characteristics, important hereditary diseases and disorders. Pak. Vet. J..

[12] Doldasheva G., Shauyenov S., Yuldashbayev Y., Ibrayev D., Mukhametzharova I. (2024). Enhancing lamb growth and meat quality: Analysis of kazakh fat-tailed and crossbred in central Kazakhstan’s sharply continental climate. Braz. J. Biol..

[13] Ombayev A., Parzhanov Z., Azhimetov N., Zhylkibayev A., Abishov M., Issabayeva A. (2023). Increasing the meat productivity of young sheep based on the use of the gene pool of the Dorper and Hissar breeds. Braz. J. Biol..

[14] Kijas J.W., Townley D., Dalrymple B.P., Heaton M.P., Maddox J.F., McGrath A., Wilson P., Ingersoll R.G., McCulloch R., McWilliam S. (2009). A genome wide survey of SNP variation reveals the genetic structure of sheep breeds. PLoS ONE.

[15] Tapio M., Ozerov M., Tapio I., Toro M.A., Marzanov N., Ćinkulov M., Goncharenko G., Kiselyova T., Murawski M., Kantanen J. (2010). Microsatellite-based genetic diversity and population structure of domestic sheep in northern Eurasia. BMC Genet..

[16] Eydivandi S., Sahana G., Momen M., Moradi M.H., Schönherz A.A. (2020). Genetic diversity in Iranian indigenous sheep vis-à-vis selected exogenous sheep breeds and wild mouflon. Anim. Genet..

[17] Jin M., Wang H., Liu G., Lu J., Yuan Z., Li T., Liu E., Lu Z., Du L., Wei C. (2024). Whole-genome resequencing of Chinese indigenous sheep provides insight into the genetic basis underlying climate adaptation. Genet. Sel. Evol..

[18] Deniskova T.E., Dotsev A.V., Selionova M.I., Kunz E., Medugorac I., Reyer H., Wimmers K., Barbato M., Traspov A.A., Brem G. (2018). Population structure and genetic diversity of 25 Russian sheep breeds based on whole-genome genotyping. Genet. Sel. Evol..

[19] Granero A., Anaya G., Demyda-Peyrás S., Alcalde M.J., Arrebola F., Molina A. (2022). Genomic population structure of the main historical genetic lines of Spanish Merino sheep. Animals.

[20] Dzomba E.F., Van Der Nest M.A., Mthembu J.N.T., Soma P., Snyman M.A., Chimonyo M., Muchadeyi F.C. (2023). Selection signature analysis and genome-wide divergence of South African Merino breeds from their founders. Front. Genet..

[21] Dossybayev K., Orazymbetova Z., Mussayeva A., Saitou N., Zhapbasov R., Makhatov B., Bekmanov B. (2019). Genetic diversity of different breeds of Kazakh sheep using microsatellite analysis. Arch. Anim. Breed..

[22] Pozharskiy A., Khamzina A., Gritsenko D., Khamzina Z., Kassymbekova S., Karimov N., Karymsakov T., Tlevlesov N. (2020). SNP genotyping and population analysis of five indigenous Kazakh sheep breeds. Livest. Sci..

[23] Dossybayev K., Amandykova M., Ualiyeva D., Kapassuly T., Kozhakhmet A., Ciani E., Bekmanov B., Amzeyev R. (2025). Genome-Wide SNP Analysis Reveals the Unique Genetic Diversity Represented by Fat-Tailed Coarse-Wooled Sheep Breeds of Kazakhstan. Biology.

[24] Iskakova Z., Alibayev N., Burabaev A., Yessirkepov M., Marzanov N. (2020). Characteristics of gene pool of various sheep breeds of the Republic of Kazakhstan. EurAsian J. BioSci..

[25] Chang C.C., Chow C.C., Tellier L.C.A.M., Vattikuti S., Purcell S.M., Lee J.J. (2015). Second-generation PLINK: Rising to the challenge of larger and richer datasets. GigaScience.

[26] Purcell S., Neale B., Todd-Brown K., Thomas L., Ferreira M.A.R., Bender D., Maller J., Sklar P., De Bakker P.I.W., Daly M.J. (2007). PLINK: A tool set for whole-genome association and population-based linkage analyses. Am. J. Hum. Genet..

[27] Zheng X., Levine D., Shen J., Gogarten S.M., Laurie C., Weir B.S. (2012). A high-performance computing toolset for relatedness and principal component analysis of SNP data. Bioinformatics.

[28] Weir B.S., Cockerham C.C. (1984). Estimating F-statistics for the analysis of population structure. Evolution.

[29] Paradis E. (2010). pegas: An R package for population genetics with an integrated-modular approach. Bioinformatics.

[30] Paradis E., Schliep K. (2019). ape 5.0: An environment for modern phylogenetics and evolutionary analyses in R. Bioinformatics.

[31] Yu G., Smith D.K., Zhu H., Guan Y., Lam T.T.-Y. (2017). ggtree: An R package for visualization and annotation of phylogenetic trees with their covariates and other associated data. Methods Ecol. Evol..

[32] Hill M.O. (1973). Diversity and evenness: A unifying notation and its consequences. Ecology.

[33] Mijangos J.L., Gruber B., Berry O., Pacioni C., Georges A. (2022). dartR v2: An accessible genetic analysis platform for conservation, ecology and agriculture. Methods Ecol. Evol..

[34] Alexander D.H., Novembre J., Lange K. (2009). Fast model-based estimation of ancestry in unrelated individuals. Genome Res..

[35] Evanno G., Regnaut S., Goudet J. (2005). Detecting the number of clusters of individuals using the software STRUCTURE: A simulation study. Mol. Ecol..

[36] Wei C., Wang H., Liu G., Wu M., Cao J., Liu Z., Liu R., Zhao F., Zhang L., Lu J. (2015). Genome-wide analysis reveals population structure and selection in Chinese indigenous sheep breeds. BMC Genom..

[37] Ciani E., Lasagna E., D’Andrea M., Alloggio I., Marroni F., Ceccobelli S., Delgado Bermejo J.V., Sarti F.M., Kijas J., Lenstra J.A. (2015). Merino and Merino-derived sheep breeds: A genome-wide intercontinental study. Genet. Sel. Evol..

[38] Ceccobelli S., Landi V., Senczuk G., Mastrangelo S., Sardina M.T., Ben-Jemaa S., Persichilli C., Karsli T., Bâlteanu V.A., Raschia M.A. (2023). A comprehensive analysis of the genetic diversity and environmental adaptability in worldwide Merino and Merino-derived sheep breeds. Genet. Sel. Evol..

[39] Megdiche S., Mastrangelo S., Ben Hamouda M., Lenstra J.A., Ciani E. (2019). A combined multi-cohort approach reveals novel and known genome-wide selection signatures for wool traits in Merino and Merino-derived sheep breeds. Front. Genet..

[40] Liu Z., Bai C., Shi L., He Y., Hu M., Sun H., Peng H., Lai W., Jiao S., Zhao Z. (2022). Detection of selection signatures in South African Mutton Merino sheep using whole-genome sequencing data. Anim. Genet..

[41] Nel C., Gurman P., Swan A., van der Werf J., Snyman M., Dzama K., Gore K., Scholtz A., Cloete S. (2022). The genomic structure of isolation across breed, country and strain for important South African and Australian sheep populations. BMC Genom..

[42] Wilson C.S., Petersen J.L., Brito L.F., Freking B.A., Nilson S.M., Taylor J.B., Murphy T.W., Lewis R.M. (2024). Assessment of genetic diversity and population structure of U.S. Polypay sheep from breed origins to future genomic selection. Front. Genet..

[43] Nilson S.M., Burke J.M., Becker G.M., Murdoch B.M., Petersen J.L., Lewis R.M. (2025). Genomic diversity of US Katahdin hair sheep. J. Anim. Breed. Genet..

[44] Addo S., Klingel S., Thaller G., Hinrichs D. (2021). Genetic diversity and the application of runs of homozygosity-based methods for inbreeding estimation in German White-headed Mutton sheep. PLoS ONE.

[45] Kooverjee B.B., Van Der Nest M.A., MacNeil M.D., Scholtz M.M., Neser F.W.C., Soma P. (2025). Estimation of breed composition of South African sheep affected with wet carcass syndrome. Front. Genet..

[46] de Sousa Silva T., Britto F.B., Sarmento J.L.R., e Silva Matias A.P.S., de Castro D.P., de Sousa F.C.B., Silva F.K.C., da Silva Santos N.P. (2025). Population structure of Santa Inês sheep: The impact of genomic information. Trop. Anim. Health Prod..

[47] Wilson C.S., Petersen J.L., Blackburn H.D., Lewis R.M. (2022). Assessing population structure and genetic diversity in US Suffolk sheep to define a framework for genomic selection. J. Hered..

[48] Puechmaille S.J. (2016). The program structure does not reliably recover the correct population structure when sampling is uneven: Subsampling and new estimators alleviate the problem. Mol. Ecol. Resour..

[49] Barani S., Nejati-Javaremi A., Moradi M.H., Moradi-Sharbabak M., Gholizadeh M., Esfandyari H. (2023). Genome-wide study of linkage disequilibrium, population structure, and inbreeding in Iranian indigenous sheep breeds. PLoS ONE.

[50] Abied A., Xu L., Sahlu B.W., Xing F., Ahbara A., Pu Y., Lin J., Berihulay H., Islam R., He X. (2020). Genome-wide analysis revealed homozygosity and demographic history of five Chinese sheep breeds adapted to different environments. Genes.

[51] Alberdi A., Gilbert M.T.P. (2019). A guide to the application of Hill numbers to DNA-based diversity analyses. Mol. Ecol. Resour..

[52] Meyermans R., Gorssen W., Aerts N., Hooyberghs K., Bhaskaran B.C., Chapard L., Buys N., Janssens S. (2024). Genomic characterisation and diversity assessment of eight endangered Belgian sheep breeds. Animal.

[53] Hall S.J.G. (2022). Genetic differentiation among livestock breeds—values for Fst. Animals.

[54] Zhao F., Deng T., Shi L., Wang W., Zhang Q., Du L., Wang L. (2020). Genomic scan for selection signature reveals fat deposition in Chinese indigenous sheep with extreme tail types. Animals.

[55] Zhu C., Li N., Cheng H., Ma Y. (2021). Genome wide association study for the identification of genes associated with tail fat deposition in Chinese sheep breeds. Biol. Open.

[56] Xu Y.X., Wang B., Jing J.N., Ma R., Luo Y.H., Li X., Yan Z., Liu Y.J., Gao L., Ren Y.L. (2023). Whole-body adipose tissue multi-omic analyses in sheep reveal molecular mechanisms underlying local adaptation to extreme environments. Commun. Biol..

[57] Kozhakhmet A., Akhatayeva Z., Dossybayev K., Yermekova M., Kapassuly T., Yergali K., Torekhanov A., Bissenov U., Lan X., Kulataev B. (2025). Genomic Characterization of the Kazakh Fat-Tailed Coarse-Wool Sheep Breed Using ROH Analysis. Animals.

[58] Jin M., Liu G., Liu E., Wang L., Jiang Y., Zheng Z., Lu J., Lu Z., Ma Y., Liu Y. (2026). Genomic insights into the population history of fat-tailed sheep and identification of two mutations that contribute to fat tail adipogenesis. J. Adv. Res..

