# Supplementary material for: Genome-Wide SNP Characterisation of Three Kazakh Sheep Breeds: Kazakh Fat-Tailed Coarse-Wool, Degeres, and Etti Merino
Source: Animals (Basel). 2026 Sep 2;16(17):2754. doi: 10.3390/ani16172754 (PMC13564707; doi:10.3390/ani16172754)
Supplement: Supplementary file 1 [file animals-16-02754-s001.zip › Supplementary_Figure_S1_breed_level_nj_tree.pdf]

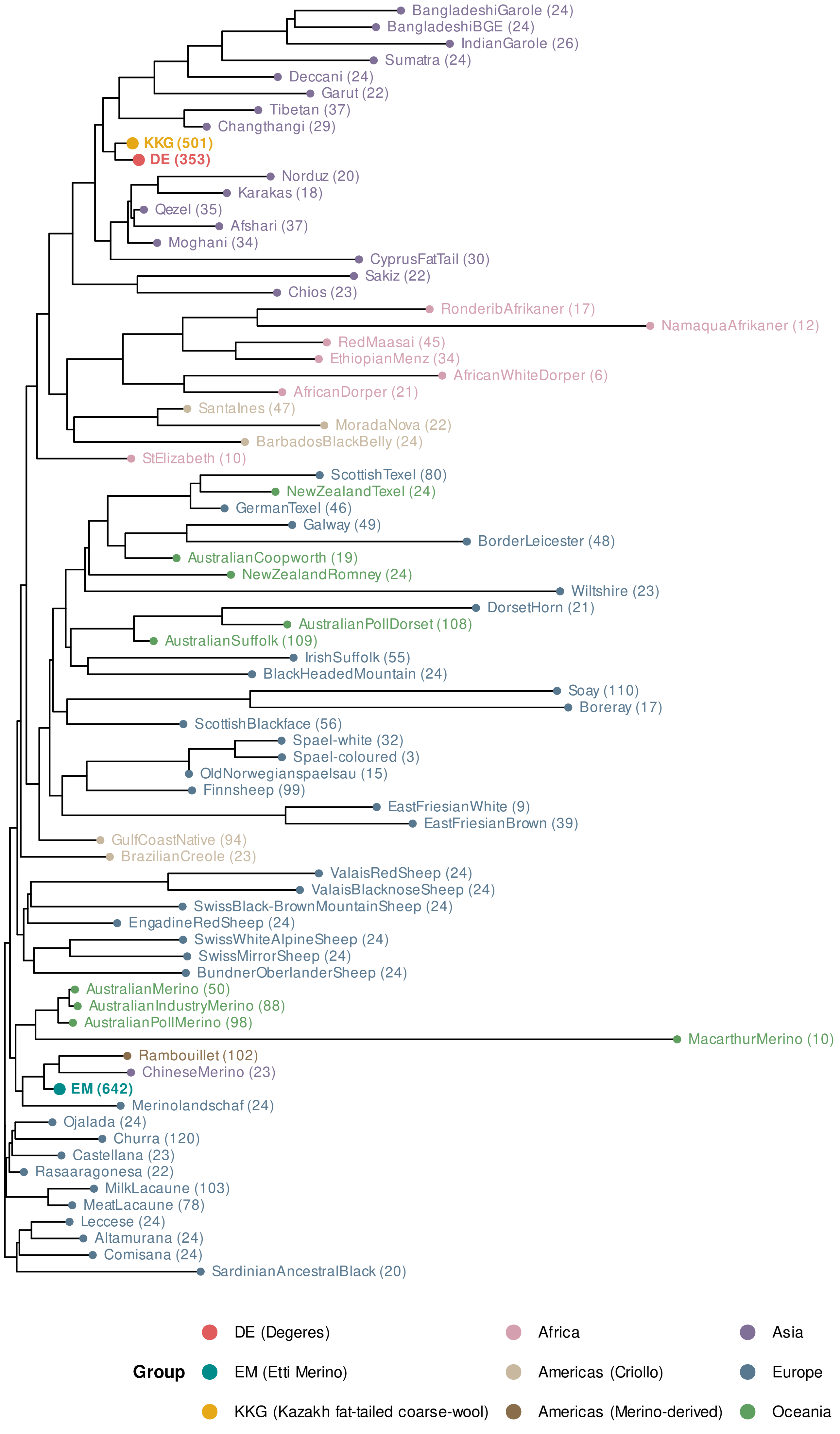
BangladeshiGarole (24)
BangladeshiBGE (24)
IndianGarole (26)
Sumatra (24)
Deccani (24)
Garut (22)
Tibetan (37)
Changthangi (29)
KKG (501)
DE (353)
Norduz (20)
Karakas (18)
Qezel (35)
Afshari (37)
Moghani (34)
CyprusFatTail (30)
Sakiz (22)
Chios (23)
RonderibAfrikaner (17)
NamaquaAfrikaner (12)
RedMaasai (45)
EthiopianMenz (34)
AfricanWhiteDorper (6)
AfricanDorper (21)
SantaInes (47)
MoradaNova (22)
BarbadosBlackBelly (24)
StElizabeth (10)
ScottishTexel (80)
NewZealandTexel (24)
GermanTexel (46)
Galway (49)
BorderLeicester (48)
AustralianCoopworth (19)
NewZealandRomney (24)
Wiltshire (23)
DorsetHorn (21)
AustralianPollDorset (108)
AustralianSuffolk (109)
IrishSuffolk (55)
BlackHeadedMountain (24)
Soay (110)
Boreray (17)
ScottishBlackface (56)
Spael-white (32)
Spael-coloured (3)
OldNorwegianspaelsau (15)
Finnsheep (99)
EastFriesianWhite (9)
EastFriesianBrown (39)
GulfCoastNative (94)
BrazilianCreole (23)
ValaisRedSheep (24)
ValaisBlacknoseSheep (24)
SwissBlack-BrownMountainSheep (24)
EngadineRedSheep (24)
SwissWhiteAlpineSheep (24)
SwissMirrorSheep (24)
BundnerOberlanderSheep (24)
AustralianMerino (50)
AustralianIndustryMerino (88)
AustralianPollMerino (98)
MacarthurMerino (10)
Rambouillet (102)
ChineseMerino (23)
EM (642)
Merinolandschaf (24)
Ojalada (24)
Churra (120)
Castellana (23)
Rasaaragonesa (22)
MilkLacaune (103)
MeatLacaune (78)
Leccese (24)
Altamurana (24)
Comisana (24)
SardinianAncestralBlack (20)
Group
DE (Degeres)
EM (Etti Merino)
KKG (Kazakh fat-tailed coarse-wool)
Africa
Americas (Criollo)
Americas (Merino-derived)
Asia
Europe
Oceania
